# *MEFV* gene methylation pattern analysis in familial Mediterranean fever patients with altered expression levels

**DOI:** 10.1186/1546-0096-13-S1-P113

**Published:** 2015-09-28

**Authors:** YZ Akkaya Ulum, B Balci Peynircioglu, ED Batu, C Guler, O Karadag, AI Ertenli, S Kiraz, S Ozen, E Yilmaz

**Affiliations:** 1 Hacettepe University, Medical Biology, Ankara, Turkey; 2 Hacettepe University, Pediatric Rheumatology, Ankara, Turkey; 3 Hacettepe University, Rheumatology, Ankara, Turkey

## Introduction

Familial Mediterranean Fever (FMF) is caused by mutations in the MEFV (Mediterranean FeVer) gene, which encodes pyrin. Phenotypic heterogeneity is very common in FMF patients and may partly rely on genetic heterogeneity. However, many cases having weak phenotypic-genotypic correlation, different clinical findings and therapeutic approaches with the same genotype show that FMF is not a simple monogenic disorder. Thus we hypothesized that possible epigenetic factors such as; DNA methylation, post-transcriptional histone modifications and microRNAs may contribute to disease phenotype in FMF patients and decided to analyze MEFV gene expression and compare the levels according to DNA methylation status in neutrophils.

## Objectives

To better understand the complexity underlying disease phenotype in FMF by the analysis of DNA methylation patterns in neutrophils and to find possible mutations in MEFV mRNA that can be related with the phenotypic heterogeneity in patients.

## Patients and methods

Blood samples were collected from 6 controls, 6 M694V/M694V patients, 6 M694V/- patients, and 6 M694V/- carriers. Neutrophil cells were isolated with Lympholyte-poly solution, Cedarlane. RNA and DNA molecules were extracted from neutrophil cells. qPCR was performed to measure MEFV gene expression levels in different individuals. For methylation analysis, DNA was treated with bisulfite by using EZ DNA Methylation-Lightning Kit, D5030, Zymo Research. Bisulfite treated DNA was amplified with special designed bisulphite primers that were specific to promoter region of the MEFV gene. DNA sequencing was performed in order to calculate the percentage of the methylation.

## Results

According to qPCR analysis, decrease in MEFV gene expression was more in homozygote group (0.12, pC and L57L were found in MEFV promoter region and exon 1, respectively (published in Infevers database). Then MEFV gene promoter region is analyzed by bisulfite sequencing in different individuals. There were no difference between patients and healthy group by means of the methylation pattern at the promoter region that we analyzed.

## Conclusion

Based on the results of this study, methylation pattern at the promoter region may not be the cause of heterogeneity for our patients with different clinical phenotype. Therefore other epigenetic mechanisms that may control MEFV expression remain to be studied.

This project is supported by Hacettepe University, Scientific Research Project Coordination Unit, Grant number: 013D05101005.

